# Potential Application of Organic Electronics in Electrical Sensing of Insects and Integrated Pest Management towards Developing Ecofriendly Replacements for Chemical Insecticides

**DOI:** 10.1002/advs.202304849

**Published:** 2023-11-09

**Authors:** Lautaro N. Petrauskas, Katherina Haase, Georg C. Schmidt, Arved C. Hübler, Stefan C. B. Mannsfeld, Frank Ellinger, Bahman K. Boroujeni

**Affiliations:** ^1^ Chair for Circuit Design and Network Theory (CCN) Faculty of Electrical and Computer Engineering Technische Universität Dresden 01069 Dresden Germany; ^2^ Chair of Organic Devices Faculty of Electrical and Computer Engineering Technische Universität Dresden 01069 Dresden Germany; ^3^ Institute for Print and Media Technology Technische Universität Chemnitz 09126 Chemnitz Germany; ^4^ Center for Advancing Electronics Dresden (cfaed) Technische Universität Dresden 01069 Dresden Germany

**Keywords:** bioelectronic sensors, nature conservation, organic field effect transistors (OFET), pesticides, printed flexible electronics

## Abstract

Synthetic insecticides are widely used against plant pest insects to protect the crops. However, many insecticides have poor selectivity and are toxic also to beneficial insects, animals, and humans. In addition, insecticide residues can remain on fruits for many days, jeopardizing food safety. For these reasons, a reusable, low‐cost electronic trap that can attract, detect, and identify, but attack only the pest while leaving beneficial insects unharmed could provide a sustainable, nature‐friendly replacement. Here, for the first time, research results are presented suggesting the great potential and compatibility of organic electronic devices and technologies with pest management. Electrical characterizations confirm that an insect's body has relatively high dielectric permittivity. Adaptive memcapacitor circuits can track the impedance change for insect detection. Other experiments show that printed polymer piezoelectric transducers on a plastic substrate can collect information about the weight and activity of insects for identification. The breakdown voltage of most insects´ integument is measured to be <200 V. Long channel organic transistors easily work at such high voltages while being safe to touch for humans thanks to their inherent low current. This feasibility study paves the way for the future development of organic electronics for physical pest control and biodiversity protection.

## Graphical abstract

This paper proposes low‐cost electronic insect traps based on organic flexible large‐area electronics for replacing harmful chemical insecticides to protect beneficial insects and natural resources. Electrical impedance characterization of several insect species, capacitive and piezoelectric sensors for detecting insects, high‐voltage organic field effect transistors (OFETs) for pest inactivation, and circuits and methods for insect detection and classification are presented.

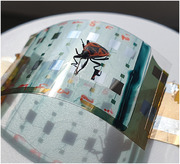

## 1 Introduction

Nature is a network of connected lives. In this network, insects provide vital ecosystem services such as pollinating the plants by carrying the pollen from one flower to another to produce new plant seeds. Around 75% of crop species that are used for human food and 94% of wild flowering plants depend on insects for pollination.^[^ ^1^ ^]^ Insects are also a major source of food for birds, amphibians, etc. Therefore, extinction or a significant decline in insect population will significantly endanger nature.

In recent decades, the biodiversity and population of insects have been threatened worldwide. Recent studies reveal dramatic rates of decline that may lead to the extinction of 40% of the world´s insect species over the next few decades.^[^ ^2^ ^]^ The meta‐analysis published by van Klink et al. reports an average worldwide decline of terrestrial insect abundance by ≈9% per decade.^[^ ^3^ ^]^ For example, a 10‐year study carried out in Germany revealed that in annually sampled grasslands, after ten years the biomass, abundance, and number of species have declined by 67%, 78%, and 34%, respectively.^[^ ^4^ ^]^ This also means that pollinator insect populations are declining, threatening both ecosystem functions and human food supplies.

The decline of insects has several major drivers such as loss of habitat due to conversion into agriculture and urbanization, massive use of agrochemicals such as insecticides and fertilizers, climate change, and biological factors such as invasive species.^[^ ^2^ ^]^

### 1.1 Insecticides and Integrated Pest Management

There is hardly any doubt that insecticides play a very important role in obtaining high crop production yields by greatly reducing the damage caused by insect pests. However, most insecticides are neuroactive, i.e., they work on the principle of attacking the insect´s nervous system.^[^ ^5^ ^]^ Although some of these chemicals are less toxic to mammals because the physiology and nervous system of mammals are different to insects, they often have poor selectivity between the insect species because they are rather similar, i.e., insecticides attack many insects including target pests, beneficial insects, natural enemies of the pest, and other non‐target insects living in the environment that are not harmful to our crops.^[^ ^6^ ^]^ As a result, the increase in insecticide use is one of the major factors causing worldwide declines in insect pollinators.^[^ ^7^ ^]^ As an example, the widely used insecticide neonicotinoid and its replacement, the sulfoximine‐based insecticide sulfoxaflor, are both harmful to bees.^[^ ^8^ ^]^

In addition to the damage to the insect population, insecticides impose other environmental challenges too. Millions of tons of these agrochemicals are released into the environment every year.^[^ ^9^ ^]^ These can be washed off by rain or wind during or after spraying and reach the soil and groundwater and contaminate them.^[^ ^10^ ^]^ They are also harmful to wild herbs and soil organisms.

Another important problem with insecticides is that residues of these chemicals can adhere to fruits and vegetable surfaces and remain there for a long time, perhaps even diffusing into them, contaminating the crops consumed as human food.^[^ ^11^ ^]^ For example, a wide variation of pesticide residues was found in the mango and guava fruits sold on the market.^[^ ^12^ ^]^ About 5‒20% of the insecticide fenvalerate is reported to remain on the Chinese mustard and cabbage 20 d after spraying, and ≈5% of the insecticide cypermethrin remains on the head lettuce after two weeks.^[^ ^13^ ^]^

Therefore, to safeguard the vital ecosystem services of insects, a serious reduction in insecticide use is urgently needed to reverse the current trends and recover insect populations. Work has been done in this direction in the nanotechnology and chemistry domains, such as targeted drug delivery into the tree´s trunk using polymeric nanocarriers, or reducing the spraying losses by enhancing pesticide droplet adhesion onto hydrophobic plant leaves by amyloid‐like aggregation of bovine serum albumin.^[^ ^14^, ^15^ ^]^ Ecofriendly biological methods are also available for some pest insects such as the mating disruption method where the synthetic sex pheromone of a specific insect is released throughout a large area to saturate the environment with the smell of that pheromone. If successful, the male insects will be confused and will not be able to locate females for mating, and in the absence of mating, only infertile eggs will be laid on the crops.^[^ ^16^, ^17^ ^]^ In this context, biopesticides are also experiencing substantial growth as a promising replacement for synthetic pesticides, e.g. biopesticides based on plant essential oils or microbial products.^[^ ^18^, ^19^ ^]^

### 1.2 State‐of‐the‐Art Electronic Traps

Electrical devices have been used in agriculture in various forms. The simple electric bug zapper, which uses a light source to attract insects and has a high‐voltage metal grid causing an electrical shock to destroy them, has been on the market for decades. However, this device has poor selectivity because light attracts many insect species including beneficial ones and they are all destroyed in the strong electric field.^[^ ^20^ ^]^ For example, the study by Frick et al. shows that only 0.22% of the insects electrocuted by electric traps in six houses in a suburban area were biting flies, while species from 104 nontarget insect families were destroyed.^[^ ^21^ ^]^ As another example of an electrical device in agriculture, Kusakari et al. have demonstrated an electric field screen that utilizes the force generated by a surface charge on an insulated electrified conductor to prevent insects from entering the interiors of facilities.^[^ ^22^ ^]^

Electronic traps equipped with high‐resolution cameras and radio transmitters are available on the market. These traps usually catch insects on sticky plates or in buckets, then take pictures and utilize image recognition algorithms in software to identify and count pest species.^[^ ^23^, ^24^ ^]^ However, it is important to note that these traps are intended for automatic monitoring of insect pest populations to help find the best time for spraying insecticides. Although these traps can help optimize/reduce the amount of insecticides applied, they are not a replacement. Monitoring requires only a few traps on the field, therefore expensive and advanced technologies can be used in monitoring traps. In contrast, large‐scale pest inactivation requires hundreds or thousands of traps distributed across the field, therefore this kind of trap, which is the topic of this study, can only be based on low‐cost technologies.

To find an electronic alternative for insecticides, Wijenberg et al. show that some insects such as German cockroaches are attracted to the electromagnetic field, therefore they propose that electrified coils as trap baits may be utilized as non‐toxic alternatives to insecticides for selective insect control in urban environments.^[^ ^25^ ^]^

It is also worth mentioning that electronic noses based on various types of aroma‐sensor technologies have been developed for the detection of plant volatiles, pesticides, insects, crop diseases, toxic gases, etc.^[^ ^26^, ^27^ ^]^ Such data can be used in integrated pest management, or these sensors may be utilized in intelligent electronic traps as well.

### 1.3 Potential Integrability of Organic Electronics into Pest Management

Organic electronics is a technological platform that offers innovative manufacturing methods for applications that do not need high‐speed computing but require mechanical flexibility and low fabrication costs. Organic devices are usually fabricated using low‐temperature solution‐processing techniques such as printing and spin coating or by thermal evaporation of small molecules and therefore are also suitable for applications where large‐area distributed circuits are required. Conventional target applications for organic electronics are radio‐frequency identification tags, electronic labels for consumer product packaging or wearables, and flexible displays, among others. In addition, the carbon‐based nature of organic materials, and the absence of heavy metals in these devices make this technology quite environmentally friendly. In previous works, we have achieved solution‐sheared printed organic semiconductor layers with reliable charge carrier mobilities up to 12 cm^2^ V^−1^ s^−1^,^[^ ^28^ ^]^ fully‐printed semi‐transparent all‐polymer organic field effect transistors (OFETs) working up to 48 V,^[^ ^29^ ^]^ an audio system consisting of an OFET amplifier and a 128 cm^2^ piezo‐polymer loudspeaker on plastic substrate reproducing sound pressure levels up to 60 dBA,^[^ ^30^ ^]^ and thermally‐evaporated vertical organic transistors with a device area as small as ≈20 µm × 20 µm and small‐signal current‐gain cutoff frequency up to *f*_T_  =  43.2 MHz at 9.8 V.^[^ ^31^ ^]^ *f*_T_ up to 160 MHz has been demonstrated by other groups at 40 V.^[^ ^32^ ^]^

**Figure 1:**
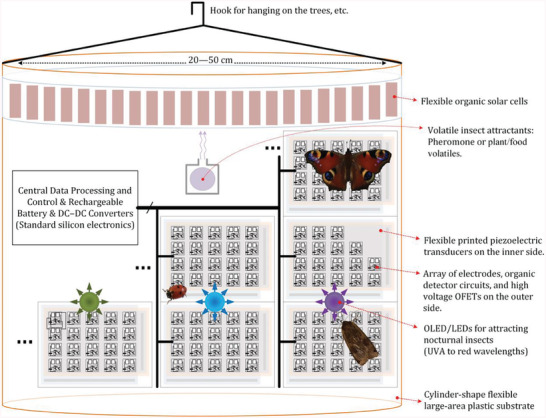
Conceptual drawing of the proposed electronic trap, based on the hybrid integration of an organic flexible large‐area analog front‐end with a compact standard‐silicon digital back‐end.

An intelligent electronic trap that works as an alternative to chemical insecticides shall be able to do the following basic functions: a) attract the insects, b) detect an insect sitting on the trap, c) identify the insect, i.e., guess with a low error rate the insect family to distinguish between the pests and nontarget insects, and d) destroy the pest while not attacking the other insects. In addition, this trap should be low‐cost, not cause pollution to the environment or the produced crops, and be electrically safe if it is accidentally touched by humans or animals such as birds. **Figure** **1** shows the proposed concept for an intelligent electronic trap based on the hybrid integration of an organic flexible large‐area analog front‐end with a compact conventional silicon‐electronic digital back‐end. The analog front‐end is responsible for collecting sensory information about the insects while the digital back‐end is responsible for control, data processing and judgment on the insects, and triggering the inactivation circuitry when a pest is detected. The fundamental principles of operation of this trap are discussed in this section. Relevant measurements on 17 insect species and high‐voltage OFETs for pest inactivation are reported in Section 2.

A key advantage of an electronic trap is that it can be reused for many years, and this could make such a device commercially viable. In contrast, farmers have to buy chemical insecticides repeatedly. In this context, it is important to note that although this electronic system requires conventional units such as a rechargeable battery, DC/DC converters, a non‐volatile memory probably in the range of 10 kB per insect species for storing the pest data, and a processor, it does not require a high‐performance digital back‐end. This is thanks to the fact that when insects sit on a surface, they typically stay there for more than one second. Therefore, we expect that an 8‒32 bit microcontroller or microprocessor with a clock frequency in the range of 10 MHz could have enough processing power for this application. We estimate average power consumption and required area for the digital back‐end to be in the range of 1 W and 100 cm^2^. However, details of the digital back‐end, required software program, communication protocol between the central processor and distributed analog front‐end, and power supply design are out of the scope of this paper and shall be studied separately. This feasibility study focuses only on the analog front end and its compatibility with organic electronic technologies. Similarly, the biological aspects of this trap shall be studied separately.

The electronic trap essentially needs to have a large‐area analog front end to be able to attract the insects and have enough space for them to fly around the trap, sit on it, walk on the surface, etc. In addition, this large‐area device should be physically non‐breakable because it is mainly intended for outdoor use. For example, if it is hung on the trees in an orchard, dust or wood particles or branches of the trees might hit it during a storm. For these reasons, organic/polymeric devices on flexible plastic substrates have promising compatibility with this application.

The first function of the electronic trap is to attract insects. Several methods are available for this purpose. Many insects such as most moths are nocturnal, i.e., they are active during the night, and many of the nocturnal insects exhibit positive phototaxis to artificial light, i.e., they get attracted towards a stimulus of light, in particular blue and ultraviolet light.^[^ ^33^ ^]^ One such example is the *codling moth* (Cydia pomonella), which is a major worldwide pest to the apple, pear, and walnut trees, and is a positive‐phototaxis nocturnal insect.^[^ ^34^ ^]^ The electronic trap can use standard LEDs or organic LEDs (OLED) for the attraction. Flexible OLEDs have been demonstrated by several groups even at a peak ultraviolet‐A wavelength of 371 nm.^[^ ^35^ ^]^ As a second method of attraction, many insects including pests are attracted to the sexual pheromones as well as plant‐derived volatile organic compounds (VOCs) such as floral aroma and fruit smell.^[^ ^36^, ^37^ ^]^ The electronic trap could release such airborne materials as shown in Figure 1. The VOC method has the advantage that it can be applied to both nocturnal and diurnal insects, but it comes with the disadvantage that the trap must be regularly refilled. In contrast, the light attraction has a big advantage that it does not need to be refilled, but it is limited to nocturnal positive‐phototaxis insects. Both methods can be nevertheless used together as complementary. A very interesting feature of light attraction is that different insect groups have different sensitivities to various light wavelengths.^[^ ^38^ ^]^ In addition, insects generally have different activity times.^[^ ^33^ ^]^ These characteristics can be utilized by the trap to enhance the selectivity. For example, when the trap is looking for a major dominant pest, it can only emit wavelengths attractive to that specific insect during its expected activity time. This can significantly minimize interference with nontarget insects.

The second and third functions of the electronic trap are to detect insects when sitting on it and to classify them with acceptable accuracy. These functions require suitable electronic sensors and circuits. In this regard, two sensors are examined in this paper. In Section 2.1 we show that when insects sit on metal electrodes like the trap´s outer surface shown in Figure 1, the electrical impedance between the two electrodes changes. This is thanks to the fact that insects are made of water, proteins, lipids, and other organic materials which have a relative dielectric constant *ε*_r_ much larger than *ε*_r‐air_ ≈ 1. Wang et al. have reported the dielectric properties of three fruit fly slurries between 27 and 1800 MHz and have measured *ε*_r_ > 40.^[^ ^39^ ^]^ However, it would be expected for an unharmed insect's dielectric constant to be smaller than those reported in the slurry, because the water‐rich internal organs are likely a large contributor to the total permittivity. In another study, radio‐frequency high dielectric losses of the insect´s eggs and larvae have been used for the postharvest thermal treatment of nuts with promising results.^[^ ^40^ ^]^ Circuits such as the one presented in Section 3.2 can track this impedance change to detect moving insect body parts coming close to the surface. The central processing unit should then collect these detection events from circuit arrays and use the data to estimate the shape, size, walking pattern, and speed of the insect sitting on the trap. This information helps to classify the insect.

For the second sensor, as shown in Figure 1, polymer‐piezoelectric transducers can be printed or attached to the inner side of the trap. When insects walk on the plastic substrate and the flexible piezo layer underneath, a vibration is produced that generates an electrical signal. The measurements in Section 2.2 show that the power spectrum of this signal is normally different for various types of insects because they have different weights, step rates, etc. This sensory information can help to better classify/guess the insect. Takahashi and Shimoyama have previously utilized piezoresistive force sensors for studying the walking and jumping of insects.^[^ ^41^ ^]^

The last function of the electronic trap is to destroy the pest insect. This can be done by applying a high voltage to some of the electrodes where the insect is sitting. Measurements in Section 2.3 show that when electrodes are in direct contact with the insect, like in Figure 1, normally a voltage below 200 V is enough to cause an electrical breakdown through the integument. In this context, we demonstrate current‐limited 200 V OFETs in Section 2.4 and further discuss suitable current source circuits bearing in mind human touch safety.

The electronic trap requires an energy source to operate, but it is intended to be used in places such as the top of the trees in the orchards where normally powering via cables is practically difficult. Therefore, the most realistic solution is to have integrated solar cells and rechargeable batteries. It is noteworthy that several flexible organic solar cells on plastic substrates with power conversion efficiency >10% have been reported in recent years.^[^ ^42^ ^]^

## 2 Experimental Results

### 2.1 Electrical Impedance Sensing of Insects

**Figure 2:**
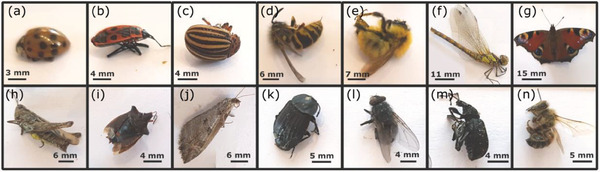
Insects studied in this paper for electrical impedance characterizations. a) Asian ladybeetle, b) Firebug, c) Colorado potato beetle, d) Common wasp, e) Common carder bee, f) Common darter, g) Peacock butterfly, h) Meadow grasshopper, i) Red‐legged shieldbug, j) Tortricid moth, k) Carrion beetle, (l) Blue bottle fly, m) Black wine weevil, n) Western honeybee. See Table S1 (Supporting Information) for species, family, and order names.

To determine the viability of detecting an insect via the impedance change over a pair of electrodes, 14 species of freshly dead insects, belonging to 7 orders and 13 families, are measured. The samples are shown in **Figure** **2**, and further biological details are given in Supporting Information Table S1 (Supporting Information). Insects were preserved at −20 °C before and after use. They were thawed before the experiments, but care was taken that no condensed water impacted the measurements.

**Figure 3:**
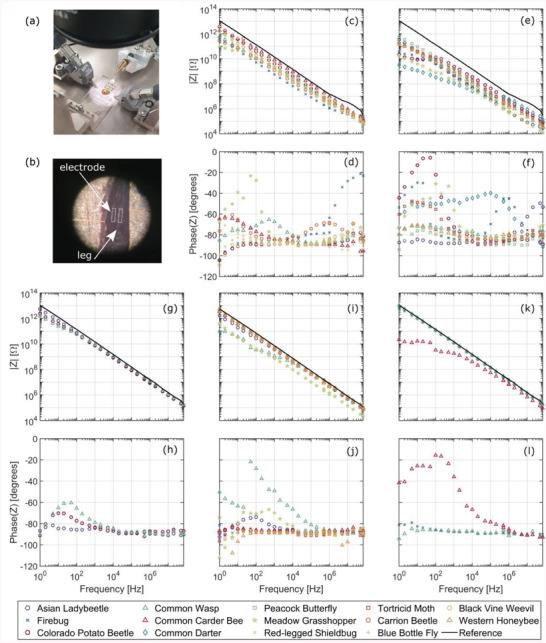
Measured insect's impedance over two electrodes. a) Electrode substrate with probe connections. b) Detailed view of an insect's femur over electrodes; in the dashed white line, we show the electrode position below the insect. Magnitude and phase of the impedance for c,d) back, e,f) abdominal, g,h) Wing, i,j) femur, and k,l) antenna body parts.

The impedance of different insects’ body parts is measured over a wide frequency range using the electrode structures and setups explained in Methods Section 5. Briefly speaking, an amplifier circuit is used for measurements at lower frequencies <1 MHz, and the S‐parameters technique for higher frequencies. **Figure** **3** presents the magnitude and phase of the impedance from 1 Hz to 70 MHz for each insect part studied, namely the back, abdominal region, wing, femur (leg), and antenna. The reference solid black line depicts the impedance when no insect is present. The electrode spacing is 1 mm for the abdominal and back, 50 µm for the femur, and 20 µm for the antenna and wing measurements.

The frequency response is mainly capacitive for most samples, dropping with a 20 dB per decade slope and having a phase close to −90°. However, at lower frequencies, the insects exhibit some resistive behavior, which can be observed from the increase in phase and flattening of the magnitude´s slope. This is compatible with the model of a lossy capacitor formed by the metallic electrodes and the insulating integument (acting as a lossy dielectric) and the water‐rich, conductive internal organs.

Particularly at lower frequencies, there is generally a big change between the impedance of loaded and unloaded electrodes. This change even reaches a factor of >1000 for the *Common darter*. Therefore, insects can be detected by impedance tracking circuits. The impedance change observed for the back, abdomen, and femur regions is larger than for the wings or antennas. This is probably because wings have a lower water content and the antenna is rather porous.

**Figure 4:**
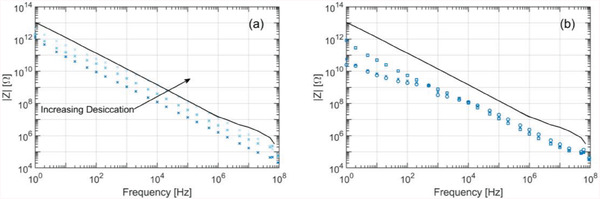
a) Effect of insect desiccation on the back‐body part impedance. b) Intra‐species variability, three firebug samples. The impedance of unloaded electrodes is depicted as a solid black line.

To better see the contribution of water content to the electrical impedance, the same freshly dead firebug is repeatedly measured after accelerating its desiccation by heating it on a hot plate at 45 °C for 60 min periods. As shown in **Figure** **4**a), the greater the desiccation, the smaller the capacitance becomes. This drying effect causes around 10 times the change of the impedance which is reasonable because water has a high *ε*_r_ ≈ 80.

In addition, to study the impedance variability within the same insect species, the abdominal sections of three freshly dead firebugs were measured and are shown in Figure 4b. It can be observed that even though they all exhibit the characteristic lossy capacitor frequency response, the magnitude of their impedance varies between the individuals, especially in the low‐frequency range. This can be attributed to different ages, genders, and metabolic states, as these might have an overall effect on the integument's thickness, the conductivity of internal organs, and water content, among others. Nevertheless, detection of an insect passing over the electrodes is possible for all measured insects because all impedances are much smaller than the unloaded electrode's impedance.

### 2.2 Piezoelectric Sensing of Insects

**Figure 5:**
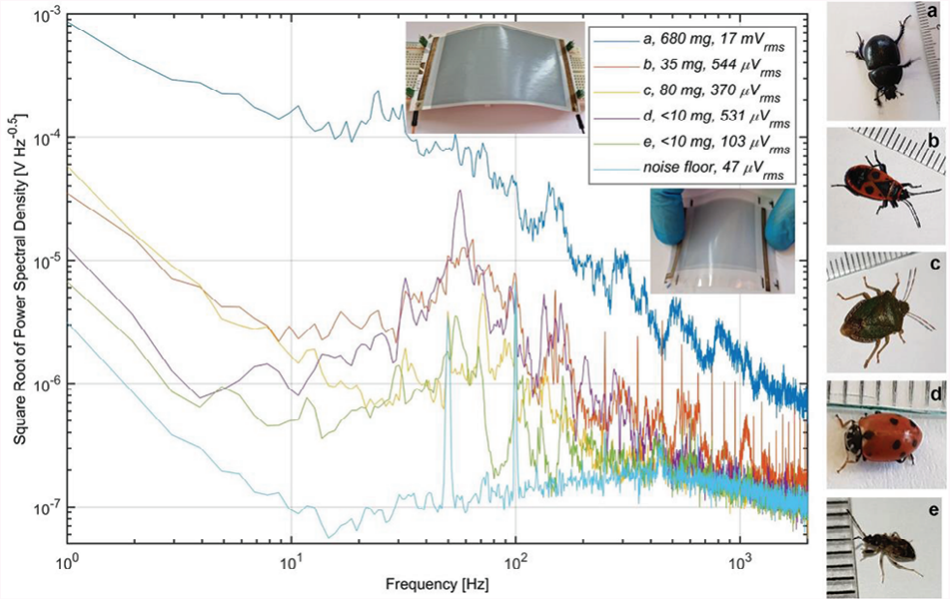
The square root of power spectral density of the voltage signal generated by the piezoelectric sensor when insects freely walk on the surface. Examples of the sensor are shown in the insets. Measured insects on the right side: a) common dor beetle, b) firebug, c) green shield bug, d) Adonis ladybird, e) dirt‐colored seed bug. The ruler is in millimeters.

Live, adult insects from five different families were placed on a printed flexible piezoelectric transducer made of piezo‐polymer Poly(vinylidene fluoride‐trifluoroethylene) (P(VDF‐TrFE)) and PEDOT:PSS conductive electrodes. Insects were not stuck to anything, they could freely walk on the surface and were released afterward. The generated electrical signal is amplified and recorded for ten seconds using the amplifier circuit and measurement setup discussed in Methods Section 5. Power spectral density (PSD) is extracted using Welch´s method in MATLAB. A typical PSD for each insect is shown in **Figure** **5**.

The most important observation is that the PSDs of different insects generally have different shapes and magnitudes. This is thanks to the fact that insects have different physical properties such as weight, step rate, step length, and walking pattern.^[^ ^43^, ^44^ ^]^ For example, the *common dor beetle* takes a few long steps per second, but in contrast, the *Adonis ladybird* takes many short steps per second, and it is much lighter than the beetle, i.e., the ladybird applies a higher rate of weaker impulses on the surface. Therefore, the spectrum of the ladybird is ≈32 times lower magnitude than the beetle, but it has a clear peak around 60 Hz. The smallest insect, i.e., *dirt‐colored seed bug*, produces the weakest signal of 103 µV_rms_ which is 165 times smaller than the large beetle. On the other side, the *green shield bug* is >8 times heavier than the ladybird, but it is still producing a weaker signal because this insect moves very slowly, i.e., it has a very low activity rate.

**Figure 6:**
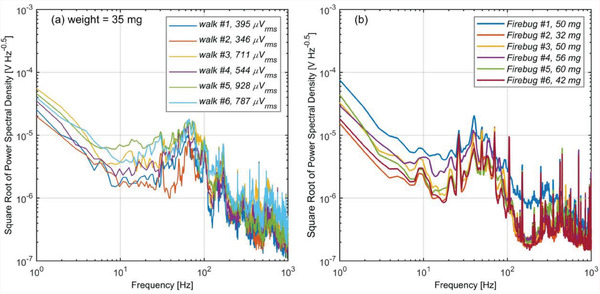
Variation of the square root of power spectral density of the voltage signal generated by the piezoelectric sensor when a) the same firebug walks on the surface six times, b) six different firebugs walk on the surface; for each firebug >5 measurements are averaged.

An insect does not walk the same way every time. Similarly, different insects from the same species might walk slightly differently. **Figure** **6**a) shows the variation of the spectrum when the same firebug is recorded six times. Figure 6b shows the spectrum of six different firebugs; here >5 recordings are averaged for each firebug. A moderate level of variation exists, but most of the spectrums more or less have similar shapes. Therefore, we expect that piezoelectric sensing is a useful tool for guessing the insect family.

Another important observation in Figure 5  is that insect movement is predominantly translated to <1 kHz as it can be seen that most PSDs reach the noise floor at 1 kHz. The electronic trap in Figure 1 requires amplifier circuits to amplify the weak piezo‐sensor signals. This is compatible with low‐speed organic transistors. For example, fully‐printed flexible transistors easily reach the current‐gain cut‐off frequency of *f*_T_  =  68 kHz and intrinsic voltage‐gain of *A*_v0_  =  43 dB which are enough for sub‐kHz signal amplification.^[^ ^29^ ^]^

It is clear that only by using piezoelectric sensing, it is not possible to distinguish between all insects. For example, there is no big difference between the firebug and the ladybird spectrums in Figure 5. For this reason, more sensors are needed in the electronic trap to reduce the probability of mistakes. Firebugs and ladybirds have different sizes and shapes that can be detected by impedance sensing on electrode arrays.

### 2.3 Electrical Breakdown of Insect Integument

To evaluate the possibility of inactivating pest insects using a high‐voltage pulse, the integument´s dielectric breakdown of the fresh dead samples shown in Figure 2 is measured. To do so, a voltage ramp from 0  to ±210 V is applied between the insect's integument outer surface and its internal organs using metallic probes, while a compliance current of 20 µA is set for safety reasons.

**Figure 7:**
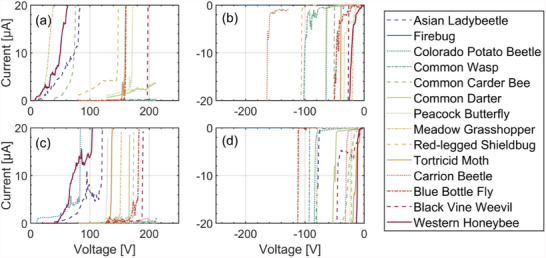
Insect's integument breakdown at positive and negative voltages for a,b) abdominal and (c,d) femur region.

As shown in **Figure** **7**, for most cases breakdown occurs below |±200 V|. Interestingly, the magnitude of the negative breakdown voltage is always lower than that of the positive breakdown voltage, though further investigation is required to understand the cause of this effect. One probable explanation could be that the outer side of the integument is quite dry while the inner side is wet. When a ΔV is applied across the integument, ions accumulate on the inner surface and electrical charges accumulate on the metallic probe touching the outer surface. Perhaps breakdown starts from the inner surface because ions can penetrate the wet tissue more easily. A positive/negative Δ*V* results in negative/positive ions on the inner surface of the integument, respectively. The penetration of positive ions such as Na^+^ might be easier than negative ions such as Cl^−^ because they are smaller or because of different diffusion mechanisms under the electric field.

As discussed in the next section, the electronic trap applies high ΔV to the pest via the electrodes on the pixel array. This would require a breakdown of the integument at two points. Therefore, the actual inactivation voltage with the system shown in Figure 1 is expected to be less or equal to the sum of the positive and negative breakdowns, i.e., in the range of 100‒400 V depending on the species. We did not have voltage sources >210 V to measure two‐point breakdowns.

It is worth mentioning that making the inactivation voltage tunable can reduce the probability of error. For example, applying 180 V to a pest that requires ≈150 V may not cause injury to nontarget insects that have a breakdown >180 V in case they have been identified in error.

### 2.4 High‐Voltage Organic Field Effect Transistors (OFETs)

As described in the previous subsection, for pest inactivation a high voltage pulse can be applied to break down the insect's integument which will then cause a lethal current passing through the conductive body. In the system concept in Figure 1, after an insect is identified as a pest, some electrodes under the pest shall be connected to a high bias voltage using transistor switches while other electrodes shall remain at a low voltage to apply a high Δ*V* to the pest´s body. Kakutani et al. have reported an electrostatic pest exclusion system and showed that a current as low as tens of microamperes can be fatal to insects.^[^ ^45^ ^]^ Still, the electronic trap should be safe if, e.g., humans or birds touch it by mistake. According to the International Electrotechnical Commission (IEC) guidelines, DC currents below 2 mA are generally not perceptible in the human body, while long‐term damage begins to occur from 25 mA onwards.^[^ ^46^ ^]^ Thus, there exists a range of currents, from tens of microamperes to ≈2 mA, which are safe for the human touch, but at the same time deadly for the pest insects.

In this regard, transistors capable of operating at hundreds of volts while delivering a limited current in the microampere range are vital for the application in mind. Both these criteria can be easily achieved with solution‐based fabrication methods. Thick polymeric dielectrics, spin‐coated or printed, can be easily tuned to the desired thickness to comply with the required high breakdown voltage. At the same time, most polymer dielectrics have a low dielectric constant which in addition to the high film thicknesses reduces the gate capacitance and as a result the current density in the channel. Furthermore, as these devices require a channel length in the range of hundreds of micrometers and low charge carrier mobility values, their fabrication can be kept at a low complexity level.

**Figure 8:**
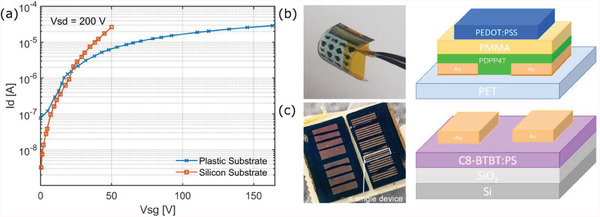
a) Transfer characteristics of two high‐voltage OFETs fabricated on rigid silicon and flexible plastic substrates. The device on silicon has *L* = 200 µm, 300 nm of SiO_2_ dielectric, and C8‐BTBT organic semiconductor. The device on PET film has *L* = 50 µm, 841 nm of PMMA dielectric, and PDPP4T polymer semiconductor. Photograph and structure of the b) plastic and c) silicon substrates with several transistors each.

Here, we present two high‐voltage organic FET examples: one over a silicon substrate and another over a plastic one. **Figure** **8** shows the transfer *I*‒*V* characteristics of these transistors. While the OFET on the silicon substrate offers a lower OFF current and exhibits an ON‐to‐OFF ratio exceeding 10^4^, it has the disadvantage of being manufactured over a rigid substrate. The plastic OFET on PET, on the other hand, can be bent and still offers an OFF current below 100 nA. The stacking technique can be used to further decrease this OFF current. An example of a high‐voltage pulse generator circuit is shown in Figure S1 (Supporting Information). It is also worth mentioning that both devices are air‐stable; the one over the silicon substrate was fabricated more than five years ago and stored under ambient conditions with neither encapsulation nor sealing. More details about the fabrication of these OFETs are given in Methods Section 5, and further discussions and examples about organic materials for outdoor applications can be found in Supporting Information and in Figure S4 (Supporting Information).

## 3 Insect Detector Circuit Concept

This section presents a detector circuit for insects based on a pinMOS organic memory element which has memory‐capacitor (memcapacitor)^[^ ^47^ ^]^ properties and low‐voltage OFETs. This opens up the possibility for the electronic trap to interact with the environment on the macro scale.

The proposed application requires a circuit that detects insects and produces a signal when they arrive on the electrodes. However, given that the circuit will be exposed to other substances, it should be able to exclude other events that could trigger the detector, e.g., water droplets falling on the surface or forming because of condensation, or dust particles. The outputs of the event detectors in the pixel array should be then collected and transmitted to the central processor, to extract information such as size, shape, movement pattern, and speed; and merge this data with the piezo‐sensor data, to distinguish between insect species and classify them either as beneficial or harmful ones.

**Figure 9:**
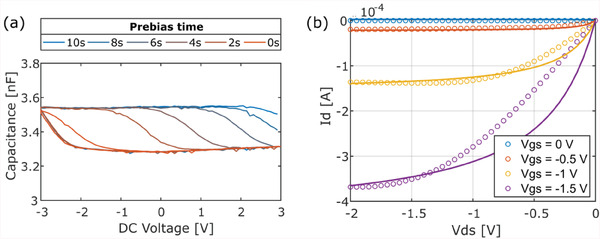
a) Measured organic pinMOS capacitance modulation for different pre‐bias lengths. b) Circle line: measured low‐voltage OFET's output characteristic. Full line: Kim model fit.

To investigate the possibility of developing such circuits with organic electronic devices, here we present one detector circuit architecture and simulate it using low‐voltage OFETs and a pinMOS organic memory element, which is a novel device introduced by Zheng et al. that behaves as a non‐volatile programmable capacitive memory.^[^ ^48^ ^]^ This feature makes it attractive to store the electrode's impedance state. This device presents a hysteresis on the *C*‒*V* curve, caused by the modulation of the depletion region in the internal p‐doped layer. It works as follows: at first glance, the pinMOS behaves similarly to a varactor—when a small DC voltage is applied between the anode and cathode, the capacitance changes as the thickness of the depletion zone gets modulated, see **Figure** **9**a in red. If, however, a pre‐bias voltage (i.e., a high‐voltage pulse before a readout operation) is applied, a tunneling current flows through the device, and charges get trapped between the P‐I‐N stack and the oxide, thus generating a new capacitive state, which can be readout by applying an AC signal. This value can be increased or decreased by changing the width, height, or polarity of the pre‐bias pulse applied, showing multi‐state storage capability as can be seen in Figure 9a.

The low‐voltage OFET is in‐house fabricated using a solution‐sheared aluminum oxide dielectric and evaporated C10‐DNTT organic semiconductor. This device presents very low off‐current in the pico‐ampere range and low threshold voltage, allowing for low‐power operation. We fitted the Kim model to the device output *I*‒*V* shown in Figure 9b. OFET fabrication details are given in Methods Section 5 and model parameters are in Table S3 (Supporting Information).^[^ ^49^ ^]^

### 3.1 Circuit Architecture

**Figure 10:**
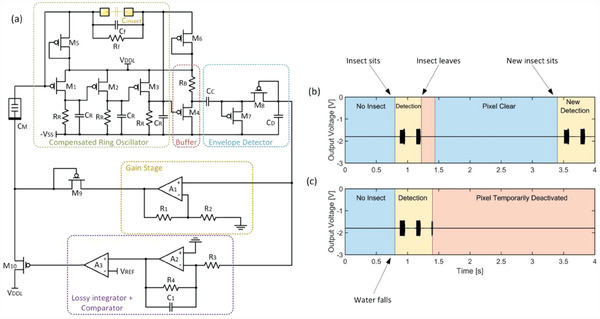
Insect detector circuit. a) Simplified block diagram. b) Simulation of the circuit for a typical event: when an insect touches the electrodes, the system triggers and generates an oscillation. If the insect moves, oscillation will stop and after a certain period highlighted in red the pixel will be ready for new detections. c) Time evolution when a water droplet falls and stays on the electrodes: the system triggers and stops after detecting it as a static object and temporarily disables the pixel.

**Figure** **10**a shows the proposed event detector architecture. The circuit exploits both the long‐term and bias‐dependent capacitance modulation of the pinMOS memory in a practical manner. Here, we call “long‐term” to the “stored” capacitive state, which is non‐volatile, and “bias‐dependent” corresponding to the “varactor effect” the device exhibits.

The circuit consists of four main blocks: a feedback‐triggered ring oscillator, a “short‐term” and a “long‐term” compensation stage, and a pinMOS memory, the latter controlled by the compensation stages.

The circuit works as follows: the ring oscillator, made up of transistors M1 to M3, is biased in such a way that it is not oscillating, but rather on the edge of oscillation, so that a small increase in capacitance takes it out of this quasi‐stable point and bring it to oscillation. This is done by having a filter on the feedback loop, consisting of Cf, Rf, and CM, which changes the feedback coefficient at one particular stage. In this way, adding any small capacitance in the loop, e.g. when an insect steps on the electrodes symbolized by Cinsect on the diagram, whatever the insect dielectric permittivity is, will increase the feedback coefficient enough to make the system start oscillating. The ring oscillator's output is passed through a buffer and then fed to an envelope detector, thus converting the AC signal to a DC voltage. Afterward, the signal is passed through a gain stage, and injected to the memory element. Therefore, after a certain time *t*_1_ passes after activation, the circuit self‐stabilizes by changing the bias of the pinMOS, acting as a normal varactor. However, if the system keeps triggering after the compensation (which would happen if any non‐moving thing such as a water droplet, dirt, or a dead insect was lying on the electrode), following a time *t*_2_>*t*_1,_ the lossy integrator stage will reach a point at which the comparator changes its state and triggers a voltage pulse that shifts the pinMOS’ long‐term capacitance until the event detector returns to the non‐oscillatory regime. In this way, the event detector adapts to the environment and forgets static activations. Completing the circuit, transistors M5 and M6 act as clamping diodes to avoid damage from the high‐voltage pulse generators by redirecting the breakdown current to the supply rail, and M9 protects the amplifier A1 from the voltage pulse generated by M10. The proposed implementation of the organic amplifiers can be found in Figure S2 (Supporting Information).

### 3.2 Circuit Simulations

To test the proposed event detector architecture, a simulation is performed on Keysight's Advanced Design System (ADS). Both the pinMOS and OFET models are written in Verilog‐A; details can be found in the Supporting Information. Figure 10b shows the time evolution of the system when an insect arrives on the electrodes at *t*  =  0.8 s and leaves shortly after. After a brief activation time, the oscillator starts to oscillate and exhibits a 600 mV peak‐to‐peak oscillation until the pinMOS changes its bias‐dependent capacitance and compensates the loop (*t*  =  0.96 s). Since the insect is still there, the short‐term capacitance relaxes and the oscillator triggers again at *t*  =  1.1 s till the insect leaves the surface. After a short refractory period marked in red the pixel is ready to detect again, which does after an insect sets over it at time *t*  =  3.4 s.

Figure 10c shows a different scenario. Here, a static stimulus such as a water droplet falling over the electrode occurs on the pixel at time *t*  =  0.8 s. After the initial detection, the oscillator tries to trigger again, but this time the long‐term capacitance‐change triggers at *t*  =  1.4 s, and the loop is compensated again at this new capacitance value. At this time, the detector requires either a larger stimulus to trigger or it will slowly return to the default operating state, whose time is set by the memory's state retention time, otherwise, it remains deactivated, avoiding subsequent triggers from the static stimulus.

To estimate the optimal power consumption required for the operation of the event detector circuit in organic technologies, the following rationale can be used. We consider an event occurring when an insect stays over the electrode for a hundred milliseconds or longer. The ring oscillator has to be able to start oscillating in this time scale, therefore, an oscillation frequency in the range of 100 Hz is needed. As each stage requires a gain of more than two in a three‐stage ring oscillator, a *f*_T_ roughly 10 times higher than the oscillation frequency for each transistor is needed. Considering the gate capacitances and loads in our circuit, transistors with a channel length of 20 µm could offer a good tradeoff between the required bandwidth and the manufacturing yield. We have previously measured the *f*_T_ as a function of power density for all‐polymer fully‐printed organic transistors with the pentacene semiconductor layer.^[^ ^29^ ^]^ For a *f*_T_  =  1 kHz at *L*  =  20 µm, the power consumption for each device is much lower than 0.1 µW mm^−1^, but here we assume this conservative value for our estimations. Additionally, in our proposed circuit, no heavy load is present at any node, allowing to shrink the transistor's width to 60 µm without detriment to the driving capabilities. Therefore, each minimum‐dimension transistor would require ≈6 nW of power. The ring oscillator then requires ≈36 nW of static power, as there is also power dissipated in the load resistors.

For each amplifier stage, however, different transistor dimensions are needed. The proposed architecture shown in Figure S2 (Supporting Information)requires only 10 transistors, but despite using larger transistors, an optimized design (i.e., smallest devices possible for the desired gain‐bandwidth‐product and load conditions) is below 900 nW. Each event detector utilizes one ring oscillator and 3 amplifiers, thus leading to a total static power below 2.8 µW.

## 4 Further Discussions and Conclusion

Since food will be a major challenge for the growing human population, developing environmentally friendly technologies for enhancing agriculture and horticulture yields while protecting nature is key to sustainable development.

In this paper, we studied the suitability of organic electronics for the detection, classification, and inactivation of pest insects, to be utilized in low‐cost electronic trap systems to replace chemical insecticides on a large scale. Organic electronics’ main limitations, namely large device size and low speed, are inconsequential for this application because of the size and timescale of insects. Yet, the low‐cost and flexible large‐area substrate compatibility of organic technologies are of great advantage for the electronic trap. However, at present, organic electronic technologies may not yet be mature enough to have good stability and yield over very large substrate areas for the complete realization of the proposed system. Fabrication process stability and yield, as well as operational stability and bias stress effects in organic transistors, shall be further improved for this purpose. In this context, it is also interesting to note that although the technology needs to be improved, the proposed application does not necessarily require a yield of 100%. Even if a few pixels are not working, the reset of the trap could continue to work and will be still a useful device but at a lower efficiency.

The impedance measurements show that detection using the insect's capacitance is possible and it dominates over the resistive losses through several frequency decades. Nonetheless, the capacitance alone does not allow for a direct classification. The power spectral density, taken from a printed piezoelectric sensor provides additional information for classification, such as weight and movement pattern. This, summed up with the insect's size, shape, and speed (which can be obtained from the electrode array), activity time and season, weather condition, and the attraction method used, should provide plenty of information to the digital back‐end to classify insects into two groups of target pests and nontarget insects with enough certainty. Chemical insecticides are blind to such information and attack all insects carelessly.

The electronic trap requires an insect database storing data such as walking patterns, size, activity time, etc. for insects living in the target region. However, the good thing is that these data are mainly needed for the pests. The trap can simply ignore, i.e., not attack, all other insects unknown to it.

The proposed trap system is divided into tiles, and each tile should have its piezo sensor and probably a printed frame around it. An area of ≈100 cm^2^ could be reasonable for each tile. Assuming a diameter and height of 20‒50 cm for the trap; 10‒70 tiles will be available to work in parallel. This trap is not expected to be infested by thousands of insects. If two or more insects are moving on one tile, the piezo‐sensor data will not be valid anymore; but this event can be detected from the electrodes array. In this case, the trap shall wait for one of the insects to move to another tile and then identify them separately. If the trap is infested by many insects, it can turn off some of the LEDs/attractants to repel some of them.

The size and spacing of electrodes also play a role in the type of detection and immunity to the environmental effects. Dust or smaller particles that could come into contact with the metallic electrode would not affect the measurement significantly if they do not cover the complete surface and therefore allow for good contact between the pad and the insect. On the other hand, other types of thick dielectric dirt appearing between two electrodes do pose an issue with detection, as they effectively lower the coupling between the electrode and insect and thus the total capacitance. Conductive materials such as plant or tree sap have the same effect as a water droplet when the electrodes are shorted and can be discriminated by the event detector. Should the conductive dirt not cover the gap between the electrodes, its effect would be minimal, as it improves the coupling between the insect surface and the electrode. Consequently, the larger the electrode distance the less sensitive to perturbations from the conditions in the field. However, this also affects the feature size of the insects that can be detected, limiting the application range. For detecting insects in the range of 1 mm or larger, pixel dimensions in the range of 200 µm × 200 µm or smaller would be required. Therefore, electrode dimensions of 50 µm × 100 µm with 50 µm spacing is a reasonable number.

If an insect carries and brings food or other substances with it to the trap, nothing will happen as long as those substances do not come close to the electrodes. But if the insect drops such substances on the surface, or if other particles accidentally fall on the trap at the same time as the insect, the electronic system may temporarily get confused because it will detect an unknown shape and shall wait for the pixels under the static substances to get disabled. Then, the trap will resume working as normal with the other pixels and can identify the insect.

It is worth noting that the insect larvae that predominantly reside within the foliage are often the ones responsible for causing significant agricultural damage. However, first, the adult female insects fly around and lay their eggs on developing fruits and foliage. These eggs then hatch into tiny larvae. If the electronic trap destroys flying pest insects, there will be fewer eggs and larvae to damage the crops.

## 5 Experimental, Fabrication, and Simulation Methods

### Impedance Measurement

**Figure 11:**
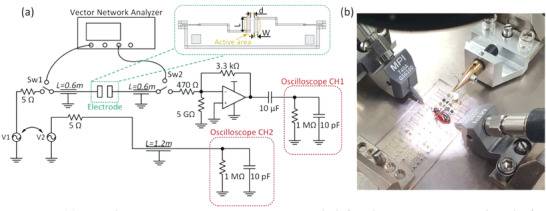
a) Impedance measurement setup. Two coupled signal generators are used to obtain the amplitude and difference phase between the reference (CH2) and the amplified insect's response (CH1). Inset: Detailed view of the electrode structure, in orange the active area is highlighted. b) Sample positioning and contacting over the substrate.

The sensor consists of two metal electrodes and two ground‐signal‐ground (GSG) pads at both ends as shown in **Figure** **11**. The distance *d* between the two electrodes in the active area varies from 1000 to 2 µm and an appropriate scaling of their length and width ensures maximum electric field through the insect body for differently sized samples. Table S2 (Supporting Information) presents the pads’ dimensions and their capacitance throughout the full measurement bandwidth (1 Hz to 70 MHz).

Insects were placed over the metallic electrodes. To maximize the sensitivity and guarantee a correct measurement, the electrode size was chosen to fit the insect part to be measured. A single test for a particular body part varied from less than a minute up to fifteen minutes, depending on the complexity of the insect positioning over the substrate.

To characterize the impedance along the full bandwidth, two different setups were utilized; a low‐frequency (1 Hz to 1 MHz) and a high‐frequency (100 kHz to 70 MHz) one, as depicted in Figure 11a (Supporting Information). The former consists of one two‐channel frequency‐coupled signal generator (Keysight Technologies 33622A, California, United States), one channel fed directly to the oscilloscope (Rhode&Schwarz RTB‐2004B, Munich, Germany), and the other signal is applied to the electrode, with an impedance conversion and gain stage connected to CH1. Before measuring insects, the cable and probe's parasitics were extracted and considered. The insect's capacitance over the electrodes is much smaller than the cable capacitance. The root‐mean‐square (RMS) voltage and phase at both oscilloscope's channels were measured 32 times and then averaged. The impedance was subsequently calculated while de‐embedding the contributions from the amplification stage.

For the high‐frequency measurement, a vector network analyzer (Rhode & Schwarz ZNB‐8A) was used. The setup was calibrated using the through‐open‐short‐match (TOSM) method with a calibration substrate. Consequently, the s‐parameters were measured and converted to y‐parameters using MATLAB's y‐parameters function from the RF toolbox. The impedance was thus calculated as the inverse of the ‐y21 parameter. A clear overlap between the low and high‐frequency setups was observed between 100 kHz to 1 MHz, proving a reliable measurement. An example is shown in Figure S3 (Supporting Information).

### EM Simulations

Electromagnetic simulations were performed using EMPro (Keysight Technologies). The substrate was modeled as a perfect insulator with a known relative permittivity of 7.5, while the metal traces were modeled by a perfect electrical conductor (PEC) with no thickness to reduce the simulation effort. The boundary conditions were absorbing. A finite‐element‐method (FEM) simulation was performed through the frequency range studied and the s‐parameters between the 2‐ports were extracted. Similarly, as with the impedance measurement, MATLAB's RF toolbox was used to convert the s‐parameters to y‐parameters and extract the structure's capacitance. Results show a match within a 6% deviation at 1 MHz with the measurements performed over an unloaded electrode (Table S2, Supporting Information).

### Fabrication of the Printed Piezoelectric Transducer

The fully printed, solution‐processed piezoelectric transducers consist of a flexible substrate, a bottom electrode, a piezoelectric polymer layer, and a top electrode. For the investigations of this work, paper sheets produced by UPM (tradename: Maxigloss) with a grammage of 90 g m^−2^ and a thickness of 67 µm were employed as lightweight and sustainable substrates. It can be noticed that polymer‐based foils (e.g., polyethylene terephthalate (PET) or polyethylene naphthalat (PEN)) can be used as semi‐transparent and environmentally more reliable alternatives, too.^[^ ^30^ ^]^ The bottom electrode, the piezoelectric polymer layer, and the top electrode were printed in sequence through screen printing using a semi‐automatic screen printer EKRA X1‐SL. The bottom and top electrodes were made of a water‐based poly(3,4‐ethylene‐dioxythiophene):poly(styrenesulfonate), PEDOT:PSS, ink (SV4, Heraeus Clevios GmbH). Before printing, the PEDOT:PSS ink was mechanically stirred for about 10 min to enhance the dispersion of the conductive polymer. Poly(vinylidene fluoride‐trifluoroethylene) (P(VDF‐TrFE)) (75:25 mol%) (FC25, Piezotech Arkema) powder was dissolved in a high‐boiling point solvent to prepare a screen printing compatible ink with good film‐leveling properties. Depending on the target layer thickness (≈5–7 µm), the co‐polymer concentration of the ink can be varied between 15 and 20% wt. After printing each layer, thermal treatment of 5 min at 130 °C (for PEDOT:PSS) or 10 min at 135 °C (for P(VDF‐TrFE)) was implemented in a box oven with air circulation to remove the solvents. This procedure resulted in a dry layer thickness of ≈500 nm and ≈6 µm for PEDOT:PSS and P(VDF‐TrFE) layers, respectively.

To get more stable and highly conductive contact pads, silver ink (Dupont 5028, Dupont Ltd., Bristol, U.K.) stripes were printed at the edges of the conductive polymer electrodes followed by thermal treatment for 5 min at 130 °C. The layer thickness of the dried silver pads was measured to be ≈5 µm.

Before using the device, electrical poling of the piezoelectric layer is required. Therefore, a high electrical field (100 MV m^−1^) was applied to align the dipoles resulting in a high remnant polarization of the co‐polymer layer in the range of ≈70 mC m^−2^. Poling was done by applying a bipolar voltage sweep that allows separation of the dipole switching from the capacitive‐charging and conduction process.^[^ ^50^ ^]^

The preparation of such flexible piezoelectric transducers can be realized using roll‐to‐roll mass printing and post‐press equipment as well.^[^ ^51^ ^]^ Such printed piezoelectric transducers can be used for different applications like loudspeakers or haptic actuators.^[^ ^50^, ^52^ ^]^

### Piezoelectricity Spectral Measurement

**Figure 12:**
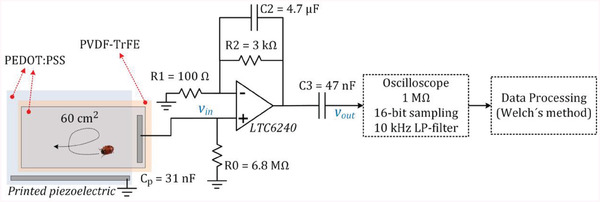
Schematic representation of the piezoelectric measurement setup of insects.

The setup is shown in **Figure** **12**. The circuit consists of an amplifier including high‐pass and low‐pass filters that amplify the signal generated by the piezoelectric transducer when an insect walks on the surface. The *R*‒*C* filters attenuate the signal power content outside of the target frequency band. This prevents mistranslation of the nontarget signal power and noise into the target measurement bandwidth. The output signal *V*_out_ is sampled for 10 s at the rate of 4k samples per second using a Rohde & Schwarz RTO2044 oscilloscope with 16‐bit resolution. The spectrum of *V*_out_ is then calculated in MATLAB using Welch's method for estimating the power spectral density (*S*_n_).^[^ ^53^ ^]^ The transfer function *H*(*jω*) from *V*_in_ to *V*_out_ is known from the *R*‒*C* values, therefore the *S*_n_ of *V*_in_ can be calculated as *S*_n_(*V*_out_)/|*H*(*jω*)|^2^. The low noise operational amplifier LTC6240 (Analog Devices Inc., USA) has a gain bandwidth product of 18 MHz and an input bias current of ≈0.2 pA. The noise from the op‐amp and resistors is negligible compared to the background noise from the environment and the piezoelectric signal.

### Fabrication of OFETs

High‐voltage OFETs were either fabricated on a rigid Si/SiO_2_ (300 nm) wafer substrate or flexible PET foil. Wafers were cleaned by subsequent sonication in water, acetone, and IPA (10 min each) followed by drying in a nitrogen stream and 10 minutes of UV/ozone exposure. Before organic semiconductor deposition, the SiO_2_ surface was treated with PTS (phenyltrichlorosilane) by immersing the cleaned wafer into a 3 wt% solution of PTS in toluene for 15 h at 90 °C. Blended semiconductor solutions with a final concentration of 4 mg mL^−1^ 2,7‐dioctyl[1]benzothieno[3,2‐b][1]benzothiophene (C8‐BTBT) in toluene and 4 mg mL^−1^ Polystyrene (PS) in anhydrous toluene were deposited by blade coating with an in‐house‐built laboratory setup. For deposition, a stage temperature of 30 °C, a blade angle of 8°, a distance between blade and substrate of 100 µm, and a coating speed of 250 µm s^−1^ were used. The BGTC devices were completed by thermal evaporation of a 50 nm thick Au source and drain electrodes.

The flexible devices were fabricated in a TGBC configuration onto cleaned (IPA, UV/ozone) PET substrates. A 2.5 nm Cr layer and a 50 nm thick Au layer were thermally evaporated through a shadow mask to define the source and drain electrodes. The commercial polymer semiconductor poly[2,5‐bis(2‐octyldodecyl)pyrrolo[3,4‐c]pyrrole‐1,4(2H,5H)‐dione −3,6‐diyl)‐alt‐(2,2′;5′,2′’;5′’,2′’’‐quaterthiophen‐5,5′’’‐diyl)] (PDPP4T) was blade‐coated at room temperature with the inhouse‐built setup from a 12 mg ml^−1^ solution in chloroform at a speed of 2 mm s^−1^. During the deposition, the blade angle was 8° and a substrate‐blade gap of 20 µm was used. The semiconductor was annealed for 20 min at 120 °C. As the dielectric, polymethyl methacrylate (PMMA) with *M*_w_ = 120 kDa dissolved in propylene glycol monomethyl ether acetate (PGMEA) at a concentration of 120 mg mL^−1^ was used. PMMA was deposited by spin‐coating at a speed of 1000 rpm for 1 min, followed by drying for 20 min at 120 °C. As the gate electrode, PEDOT:PSS was used which was applied with a brush and dried for 5 min at 80 °C and a further 10 min at 120 °C.

Low‐voltage OFETs were prepared on cleaned Si wafers. After 20 min of UV/ozone exposure, a precursor solution (0.2 m aluminum nitrate nonahydrate in di‐ionized water) was shear‐coated with a speed of 10 mm s^−1^, a stage temperature of 80 °C, blade‐substrate gap of 20 µm and blade angle of 8°. The deposited film was annealed for 5 min at 300 °C. To obtain a layer thickness of about 20 nm, 5 subsequent layers were deposited. Afterward, the dielectric surface was coated with Tetradecylphosphonic acid (TDPA) by immersing the substrates overnight into a 5 × 10^‐3^ m solution in IPA. After immersion, the substrates were rinsed with IPA and baked for 5 min at 60 °C. The organic semiconductor 2,9‐didecyldinaphtho[2,3‐b:2′,3′‐f]thieno[3,2‐b]thiophene (C10‐DNTT) was then deposited in a high vacuum at a deposition rate of 0.2 Å s^−1^ to a thickness of about 25 nm. Finally, 50 nm thick Au source and drain electrodes were deposited through thermal evaporation.

### OFET Modeling

Following the OFET fabrication, their transfer and output characteristics were obtained and recorded. Consequently, the OFET´s DC behavior was fit to the compact model proposed by Kim et al.,^[^ ^49^ ^]^ while the AC characteristics were extrapolated from the capacitances measured on test structures. The fitting parameters can be found in Table S3 (Supporting Information). The model was then written in Verilog‐A and verified using Keysight's ADS.

### Memcapacitor Modeling

The memory element was simulated using a Verilog‐A model on Keysight's ADS. The model consists of a varactor in series with a capacitor. Thus, the total capacitance is given by the series connection of the varactor's and capacitor's capacitance. The former, which can be modulated by the applied bias, is expressed as a sigmoid function with a given offset. The long‐term capacitance, on the other hand, is stepped by voltage‐controlled voltage sources (VCVS), which depending on the applied bias connects or disconnects further capacitors in parallel to the main branch.

## Author Contributions

B.K.B. proposed the application of organic electronics in pest management and the trap´s sensors and system concept. B.K.B., S.C.B.M., and F.E. conceived and designed the project. The pinMOS memcapacitor was designed by the team of S.C.B.M. B.K.B. and L.N.P. designed the experiments. L.N.P. performed impedance and breakdown measurements as well as EM simulations with the help of B.K.B. L.N.P. performed device modeling and detector circuit design and simulation while B.K.B. proposed the oscillator and F.E. supervised this work. B.K.B. and L.N.P. performed piezoelectricity measurements using transducers designed and printed by G.C.S and A.C.H. OFETs were designed and fabricated by K.H. and S.C.B.M.

## Conflict of Interest

The authors declare no conflict of interest.

## Supporting information

Supporting Information

## Data Availability

The data that support the findings of this study are available from the corresponding author upon reasonable request.
