# Supplementary material for: Potential Application of Organic Electronics in Electrical Sensing of Insects and Integrated Pest Management towards Developing Ecofriendly Replacements for Chemical Insecticides
Source: Adv Sci (Weinh). 2023 Nov 9;11(4):2304849. doi: 10.1002/advs.202304849 (PMC10811475; doi:10.1002/advs.202304849)
Supplement: Supplementary file 1 — Supporting Information [file ADVS-11-2304849-s001.pdf]

ADVANCED SCIENCE
Open Access

Supporting Information

for *Adv. Sci.*, DOI 10.1002/advs.202304849

Potential Application of Organic Electronics in Electrical Sensing of Insects and Integrated Pest Management towards Developing Ecofriendly Replacements for Chemical Insecticides

*Lautaro N. Petrauskas, Katherina Haase, Georg C. Schmidt, Arved C. Hübler, Stefan C. B. Mannsfeld, Frank Ellinger and Bahman K. Boroujeni**

Supporting Information

Potential Application of Organic Electronics in Electrical Sensing of Insects and Integrated Pest Management Towards Developing Ecofriendly Replacements for Chemical Insecticides

*Lautaro N. Petrauskas, Katherina Haase, Georg C. Schmidt, Arved C. Hübler, Stefan C. B. Mannsfeld, Frank Ellinger, and Bahman K. Boroujeni**

Table S1. Insect species used in this study, their assignment to orders, families, trivial names, and references in this publication.

Species	Order	Family	Trivial name	Reference
Harmonia axyridis Pallas	Coleoptera	Coccinellidae	Asian ladybeetle	Fig. 2A
Pyrrhocoris apterus L.	Heteroptera	Pyrrhocoridae	firebug	Fig. 2B
Leptinotarsa decemlineata Say	Coleoptera	Chrysomelidae	Colorado potato beetle	Fig. 2C
Vespula vulgaris L.	Hymenoptera	Vespidae	common wasp	Fig. 2D
Bombus pascuorum Scop.	Hymenoptera	Apidae	common carder bee	Fig. 2E
Sympetrum striolatum Charp.	Odonata	Libellulidae	common darter	Fig. 2F
Aglais io L.	Lepidoptera	Nymphalidae	peacock butterfly	Fig. 2G
Chorthippus parallelus Zett	Orthoptera	Acrididae	meadow grasshopper	Fig. 2H
Pentatoma rufipes L.	Heteroptera	Pentatomidae	red-legged shieldbug	Fig. 2I
Ancylis achatana (Denis & Schiffermüller)	Lepidoptera	Tortricidae	tortricid moth	Fig. 2J
Silpha tristis Ill.	Coleoptera	Silphidae	carrion beetle	Fig. 2K
Calliphora vicina Rob.-Des.	Diptera	Calliphoridae	blue bottle fly	Fig. 2L
Otiorhynchus sulcatus Fab.	Coleoptera	Curculionidae	black wine weevil	Fig. 2M
Apis mellifera L.	Hymenoptera	Apidae	western honeybee	Fig. 2N
Geotrupes stercorarius L.	Coleoptera	Geotrupidae	common dor beetle	Fig. 5a
Pyrrhocoris apterus L.	Heteroptera	Pyrrhocoridae	firebug	Fig. 5b

Palomena prasina L.	Heteroptera	Pentatomidae	green shield bug	Fig. 5c
Hippodamia variegata Goeze	Coleoptera	Coccinellidae	Adonis ladybird	Fig. 5d
Rhyparochromus vulgaris Schilling	Heteroptera	Rhyparochromidae	dirt-colored seed bug	Fig. 5e

Table S2. Electrodes fabricated for the impedance measurements, their dimensions, and measured versus EM-simulated capacitance.

d [um]	W [um]	L [um]	Measured Capacitance [fF]	Simulated Capacitance [fF]
1000	1000	1000	10.3	9.8
500	500	1000	22.0	21.9
200	200	800	28.6	30.3
100	100	800	22.8	24.6
50	50	400	25.2	26.4
20	20	160	14.0	14.9

Table S3. Model parameters used for the low-voltage OFET model, based on the Kim model. **Error! Reference source not found.**

Parameter	Unit	Value
γ	-	0.226
Vt	V	-0.142
μo	$cm^2 V^{-1} s^{-1}$	1.91
Vo	V	-0.14
α	-	0.46
Rc	Ω	100
m	-	1.05
λ	V^{-1}	0
S	$Vdec^{-1}$	0.515
Vb	V	0.9
B	V^{-1}	0.043
Cox	$F m^{-2}$	0.0021
Vaa	V	0.91
Ioff	pA	100

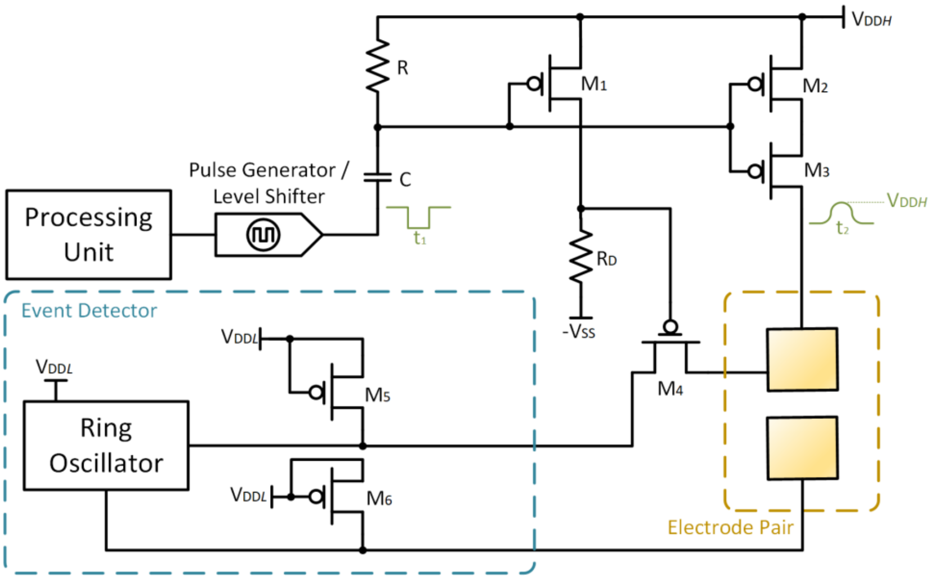

Figure S1. An example of a high-voltage pulse generation circuit. When inactive, transistors M1–M3 remain OFF, thus the electrode is disconnected from the high-voltage V_{DDH}, but M4 is ON, making a low-impedance connection between the electrode and the event detector circuitry for sensing. When the active-low input pulse arrives, M1–M3 turn on. Consequently, the voltage V_{DDH} appears on one of the electrodes, injecting a breakdown current into the pest. This current will be discharged to the V_{DDL} through the internal clamp diodes (M5 and M6) in the other pixels touching the other legs of the pest. During this time, the low-voltage circuitry is protected from the high voltage by M4, which remains OFF during the pulse. This circuit has two layers of protection for human touch safety: a) OFET I_{ON} is < 2 mA, b) the high-voltage pulse width is limited by the R–C time constant and cannot exceed a certain duration, i.e. $t2 \leq t1$.

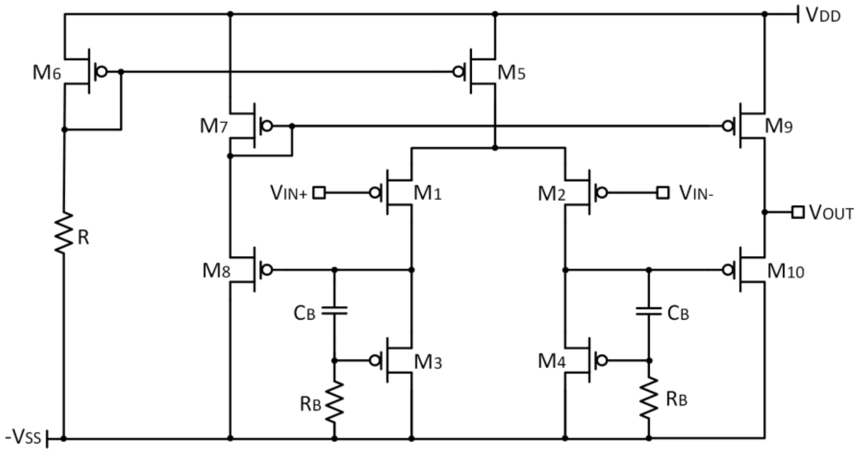

Figure S2. Proposed organic differential to single-ended amplifier. The core consists of the transistors M1–M5. M1 and M2 perform the voltage-to-current conversion, while M3 and M4 act as the loads. C_B and R_B form a high-pass filter between the source and gate, thus increasing

the gain (bootstrapping). M6 and M5 make up a current mirror, which biases the differential core. The circuit gets completed by M7–M9, acting as source followers and increasing the total gain without strongly loading the differential input pair.

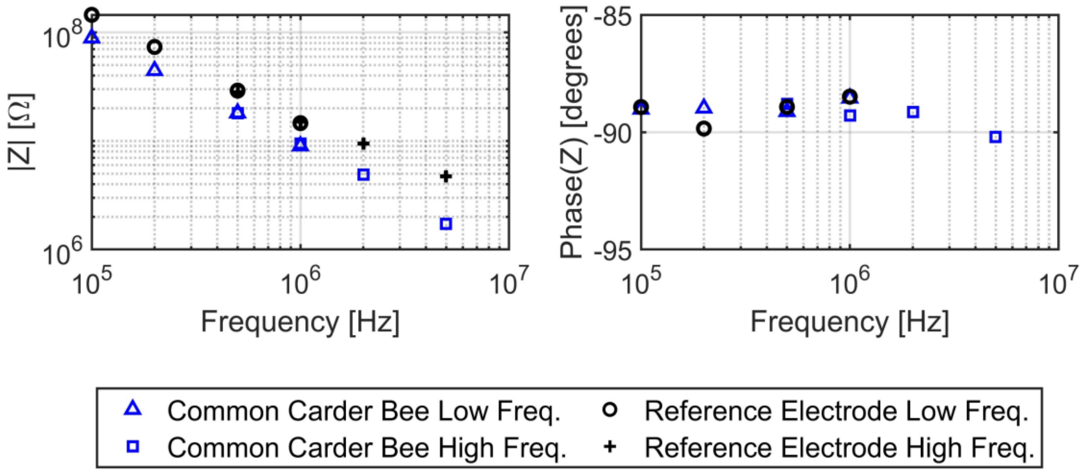

Figure S3. Examples of matching between the low-frequency and high-frequency impedance measurement setups at 500 kHz and 1 MHz.

Organic electronic materials for outdoor applications

While organic electronics provide several advantages for the proposed outdoor application, such as their large-area fabrication capability, transparency, and mechanical flexibility, organic electronics materials are also known to have poor operational stability, especially in ambient conditions when exposed to light, moisture, oxygen, or temperature variations.

However, in recent years, great progress has been made in the enhancement of these deficits pushing the technologies further into commercialization.[1] Such strategies range from molecular design strategies, over fabrication-oriented approaches, to the use of barrier films. [2,3]

As for the dielectric materials, the use of amorphous fluoropolymers has proven to be a suitable approach for enhancing the environmental robustness of field-effect transistors.[4] This class of materials is printable, provides good barrier characteristics, and can be employed to realize gate dielectrics in high-voltage OFETs that simultaneously act as barrier layers. Furthermore, in previous work, we could show that our high-capacitance, shear-coated polymer dielectric – a system that could serve to realize low-voltage OFETs for the detector circuit – shows stable performance when cycled 1000 times in ambient conditions.[5] Moreover, it was demonstrated that this material system can also operate in aqueous media.[6] Even the AlOx dielectric used here with the hydrophobic interface modification (TDPA) in combination with the stable

organic semiconductor C10-DNTT provides excellent operational stability in ambient conditions, which has been also reported by other groups.[7] However, the possibility of encapsulating those devices against mechanical stress and excess water should be studied in the future.

Concerning the semiconducting layer, a range of approaches are currently available to provide good ambient stability. Specifically, the development of p-type/n-type materials with lowered HOMO/LUMO levels and large bandgap represents a promising strategy. For instance, iso-indigo-based materials with low-lying HOMO levels have been developed, and it was shown that such semiconductors have excellent air stability.[8] Chemical design strategies were also applied to n-type materials providing improved stability in ambient conditions.[9] While in this work, we have used the polymer semiconductor PDPP4T with a HOMO level of about -5.2 eV, but a relatively small bandgap of 1.2 eV, we are currently working on the realization of flexible iso-indigo-based, high-voltage OFETs. Furthermore, as previously mentioned, the (fabricated PDPP4T) devices' stability could be enhanced by replacing the PMMA dielectric with other polymer insulators with lower oxygen and water permeability.

Here, high-voltage OFETs were further prepared with the small molecule semiconductor C8-BTBT. With its low-lying HOMO level and large bandgap, it exhibits excellent air stability, which is also seen for other related compounds. In this regard, dinaphtho[2,3-b:2′,3′-f]thieno[3,2-b]thiophene (DNTT) and its derivatives are very promising candidates.[7] Additionally, in our work we could enhance the stability through the blending of the inert, hydrophobic polymer polystyrene (PS), which is by now a rather universal strategy[10] that was successfully applied to other semiconductors, such as the semiconducting copolymer, poly(didodecylquaterthiophene-alt-didodecylbithiazole) (PQTBTz-C12).[11] In the future, materials with specific functional groups[12] attached to the semiconducting core, doping,[13] as well as interface engineering might be explored, all of which are strategies that are proven to provide good stability in the presence of diverse environmental factors.

A general and very effective strategy to minimize degradation upon extrinsic factors is the introduction of barrier layers. In this regard, spin-coated polymer layers[8], amorphous fluoropolymers as well as metal-oxides[14] have been proven to be very effective.

In summary, a broad spectrum of opportunities for the design and fabrication of robust organic devices exists. For this particular application case, some of the strategies are already applied with promising results, an example is shown in Figure S4. Hence, by incorporating further strategies for the enhancement of environmental robustness, the advantages of organic electronics will open up new applications such as the proposed electronic trap.

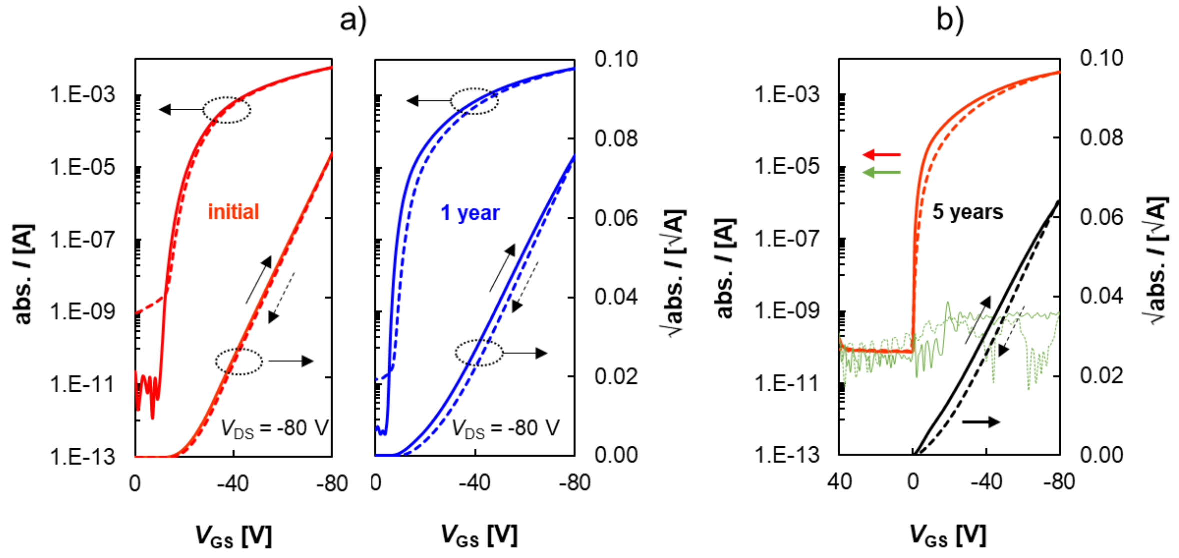

Figure S4. Demonstration of ambient stability of C8-BTBT:PS-based OFET: a) same device measured after fabrication and after one year of storage; b) device on the same sample measured after 5 years storage in ambient conditions.

References of Supporting Information

1. Chen, Y., Deng, W., Zhang, X., Wang, M. & Jie, J. Ambient instability of organic field-effect transistors and their improvement strategies. *J. Phys. D. Appl. Phys.* **55,** (2022).
2. Lee, E. K., Lee, M. Y., Park, C. H., Lee, H. R. & Oh, J. H. Toward Environmentally Robust Organic Electronics: Approaches and Applications. *Adv. Mater.* **29,** 1–29 (2017).
3. Kimpel, J. & Michinobu, T. Conjugated polymers for functional applications: lifetime and performance of polymeric organic semiconductors in organic field-effect transistors. *Polym. Int.* **70,** 367–373 (2021).
4. Roh, J., Cho, I.-T., Shin, H., Woo Baek, G., Hee Hong, B., Lee, J.-H., Hun Jin, S. & Lee, C. Fluorinated CYTOP passivation effects on the electrical reliability of multilayer MoS 2 field-effect transistors. *Nanotechnology* **26,** 455201 (2015).
5. Haase, K., Zessin, J., Zoumboulis, K., Müller, M., Hambsch, M. & Mannsfeld, S. C. B. Solution Shearing of a High-Capacitance Polymer Dielectric for Low-Voltage Organic Transistors. *Adv. Electron. Mater.* **5,** 1900067 (2019).
6. Roberts, M. E., Mannsfeld, S. C. B., Queralto, N., Reese, C., Locklin, J., Knoll, W. & Bao, Z. Water-stable organic transistors and their application in chemical and biological sensors. *Proc. Natl. Acad. Sci.* **105,** 12134–12139 (2008).

7. Zschieschang, U., Klauk, H., Kang, M. J., Takimiya, K., Sekitani, T., Someya, T., Canzler, T. W., Werner, A. & Blochwitz-Nimoth, J. Performance and stability of flexible low-voltage organic thin-film transistors based on C<inf>10</inf>-DNTT. in *2012 8th Int. Caribb. Conf. Devices, Circuits Syst.* 1–4 (IEEE, 2012). doi:10.1109/ICCDCS.2012.6188911
8. Lei, T., Cao, Y., Fan, Y., Liu, C., Yuan, S. & Pei, J. High-Performance Air-Stable Organic Field-Effect Transistors: Isoindigo-Based Conjugated Polymers. *J. Am. Chem. Soc.* **133,** 6099–6101 (2011).
9. Fujiseki, T., Fujimoto, S., Campoy-Quiles, M., Alonso, M. I., Murakami, T. N., Miyadera, T. & Fujiwara, H. in *Springer Ser. Opt. Sci.* **214,** 427–469 (2018).
10. Riera-Galindo, S., Leonardi, F., Pfattner, R. & Mas-Torrent, M. Organic Semiconductor/Polymer Blend Films for Organic Field-Effect Transistors. *Adv. Mater. Technol.* **4,** (2019).
11. Kwak, D., Choi, H. H., Kang, B., Kim, D. H., Lee, W. H. & Cho, K. Tailoring Morphology and Structure of Inkjet-Printed Liquid-Crystalline Semiconductor/Insulating Polymer Blends for High-Stability Organic Transistors. *Adv. Funct. Mater.* **26,** 3003–3011 (2016).
12. Cho, Y., Sun, Z., Lee, K. M., Zeng, G., Jeong, S., Yang, S., Lee, J. E., Lee, B., Kang, S.-H., Li, Y., Li, Y., Kwak, S. K. & Yang, C. CF 3 -Terminated Side Chain Enables Efficiencies Surpassing 18.2% and 16.1% in Small- and Large-Scale Manufacturing of Organic Solar Cells. *ACS Energy Lett.* **8,** 96–106 (2023).
13. Ghamari, P., Niazi, M. R. & Perepichka, D. F. Improving Environmental and Operational Stability of Polymer Field-Effect Transistors by Doping with Tetranitrofluorenone. *ACS Appl. Mater. Interfaces* **15,** 19290–19299 (2023).
14. Kim, S. H., Yoon, W. M., Jang, M., Yang, H., Park, J.-J. & Park, C. E. Damage-free hybrid encapsulation of organic field-effect transistors to reduce environmental instability. *J. Mater. Chem.* **22,** 7731 (2012).
